# Significant Acute Kidney Injury Due to Non-steroidal Anti-inflammatory Drugs: Inpatient Setting

**DOI:** 10.3390/ph3041279

**Published:** 2010-04-26

**Authors:** Mehul Dixit, Thuy Doan, Rebecca Kirschner, Naznin Dixit

**Affiliations:** Florida Children’s Kidney Center, Department of Nephrology, University of Central Florida College of Medicine, 615 E. Princeton Suite 500 Orlando, FL 32803, USA

**Keywords:** children, pain killers, renal insufficiency, ibuprofen, dialysis, steroid, acute interstitial nephritis, acute renal failure

## Abstract

In the United States non-steroidal anti-inflammatory drugs (NSAID) are freely available over-the-counter. Because of the adverse effects on the kidneys and the popularity of these drugs, unregulated use of NSAIDs is an under recognized and potentially dangerous problem. Fifteen inpatients, mean age of 15.2 ± 2.3 years (five males, 10 females), were referred to nephrology for acute kidney injury. All patients admitted to taking ibuprofen and six also consumed naproxen. None of the patients had underlying renal diseases at the time of admission. Nine patients had proteinuria and 12 had hematuria (including one with gross hematuria). One patient had nephrotic syndrome but the condition resolved spontaneously without steroids and has remained in remission for four years. Two patients required dialysis. Only one of the dialyzed patients required steroid therapy for recovery of renal function. The mean duration of hospitalization was 7.4 ± 5.5 days. The serum creatinine peaked at 4.09 ± 4.24 (range 1.2-15.3) mg/dL. All patients recovered renal function with normalization of serum creatinine to 0.71 ± 0.15 mg/dL. The estimated GFR (glomerular filtration rate) at peak of renal failure was 38.2 ± 20.5 mL/min but did improve to a baseline of 134 ± 26.2 mL/min (range 89-177, p < 0.01). However, the duration from onset to normalization of serum creatinine was 37 ± 42 days indicating that majority of patients had abnormal renal function for a prolonged period. In conclusion, NSAIDs pose a significant risk of renal failure for significant duration and as an entity may be under recognized.

## 1. Introduction

The kidneys receive approximately 25% of the cardiac output and are the major organ for drug excretion [1]. Due to this function, the renal arterioles and glomerular capillaries are especially vulnerable to the effects of drugs [1]. Non-steroidal anti-inflammatory drugs (NSAIDs) are one of the most commonly used over-the-counter (OTC) medications in the United States and are known to have adverse effects on kidney function [2]. OTC NSAIDs, including ibuprofen, are routinely administered to children or taken by teenagers for pain and fever [3]. Use of NSAIDs has increased dramatically during recent years, especially in children in the United States [4]. Because of their frequent and accepted use, NSAIDs are widely considered safe, but in reality, even therapeutic doses carry a risk of loss of renal function [2].

Adverse renal effects from these drugs are caused by two distinct pathological entities. The first mechanism of acute kidney injury (AKI) from NSAIDs is due to reduced renal plasma flow caused by a decrease in prostaglandins, which regulate vasodilation at the glomerular level. NSAIDs disrupt the compensatory vasodilation response of renal prostaglandins to vasoconstrictor hormones released by the body [5]. Inhibition of renal prostaglandins results in acute deterioration of renal function after ingestion of NSAIDs. The second mechanism of AKI is acute interstitial nephritis (AIN), which is characterized by the presence of an inflammatory cell infiltrate in the interstitium of the kidney. AIN is caused by an immunological reaction after NSAID exposure of about a week [6,7,8]. AIN is now recognized as a major cause of drug induced AKI and accounts for about 15% of all patients with unexplained AKI [7]. As an entity, AIN due to NSAIDs is under recognized as well. 

We describe fifteen inpatients with significant renal dysfunction due to NSAIDs. The spectrum of clinical syndrome included prolonged elevation of creatinine, nephrotic syndrome in one, dialysis, and steroid therapy. In hospitalized patients with unexplained renal failure, administration of NSAIDs should be explored. This study is not designed to estimate true prevalence of NSAIDs induced AKI, but is rather more a descriptive analysis of referred cases.

## 2. Patients and Methods

Medical records of fifteen inpatients aged 9 to 19 years (mean age of 15.2 ± 2.3 years, five males and 10 females) who were referred to Pediatric Nephrology service for acute kidney injury (AKI) at the University Medical Center, Tucson (13 cases), and the Florida Children’s Hospital, Orlando (2 cases), between the periods of 2003 to 2008 were reviewed.

An evaluation by a pediatric nephrologist regarding the etiology of renal failure was carefully documented. Any patient with documented intrinsic renal failure due to glomerulonephritis, nephrotic syndrome, obstructive renal disease, infective renal disease, septic shock, hemodynamic instability, or multi-organ failure were excluded. Documented evidence of intrinsic renal failure due to NSAIDs excluding any other cause of AKI was deemed satisfactory for inclusion.

The mean age of males was 13 ± 3.3 years and the mean age of females was 16 ± 1.3 years. The patients’ charts were reviewed for all available data. A detailed past history, including family history of renal diseases, current medications, and any over-the-counter medications administered prior to admission were reviewed. Urinalysis was documented with special note of the presence of hematuria (gross and microscopic) and proteinuria defined as detectable on a dipstick or by abnormal 24 hour urine, if one was available. The serial laboratory analysis of renal function was noted. The glomerular filtration rate (eGFR) was calculated using Schwartz’s formula [9]. Kidney biopsy results on patients who underwent the procedure were noted. Duration of admission, serial laboratory data including renal function, urinalysis, and blood pressure at the time of discharge and subsequent followup were noted. The treatment administered was reviewed. Special note of dialysis therapy requirement and administration of steroid therapy was made. The length of duration of normalization of creatinine and last available outpatient follow-up were included in data analysis.

Results are expressed as mean and standard deviation (± SD). A student’s t-test between the peak creatinine and baseline creatinine as well as between the eGFR and baseline eGFR was done. A *p* value of <0.05 was considered statistically significant.

## 3. Results

Table 1 describes the clinical characteristics of the fifteen patients. None of the fifteen patients had underlying renal diseases at the time of admission. All patients reported that they ingested recommended doses of NSAIDs. Nine patients had proteinuria and 12 had hematuria including one patient with gross hematuria. As shown in Table 2, the range of serum creatinine was 1.2-15.3 mg/dL (mean mg/dL 4.09 ± 4.24) at the peak of renal failure. The mean eGFR at peak of renal failure was 8.2 ± 20.5 mL/min.

**Table 1:** Clinical parameters of 15 inpatients with NSAID induced AKI**.**

Patient	Age yrs	Sex	Flank Pain	Oliguria	Hypertension	Proteinuria	Hematuria	Biopsy
1	19	F	Y	N	N	N	Y	N
2	14	M	Y	N	N	N	Y	N
3	14	F	Y	N	N	Y	N	N
4	18	F	Y	N	N	Y	Y	N
5	16	F	Y	N	N	Y	Y	Y
6	15	F	Y	N	N	N	Y	N
7	14	F	Y	N	N	Y	N	Y
8	17	F	Y	N	N	N	Y	N
9	16	F	Y	N	N	Y	Y	Y
10	15	M	Y	N	Y	Y	Y	Y
11	16	F	Y	N	N	Y	Y	N
12	9	M	Y	N	N	Y (nephrotic)	Y	Y
13	10	M	Y	N	Y	Y	Y	N
14	17	F	Y	N	N	N	Y	N
15	17	M	Y	N	N	N	N	N

### 3.1. Nephrotic syndrome

Only one out of the fifteen patients had nephrotic range proteinuria. He had symptomatic nephrotic syndrome with anasarca, hypoalbunemia, and hypercholesteremia. The kidney biopsy showed minimal change disease with AIN. The patient had spontaneous resolution of nephrotic syndrome within two weeks without steroid therapy. Four years later with avoidance of NSAIDs, he has not had a relapse of nephrotic syndrome.

### 3.2. Kidney Biopsy

Five out of fifteen patients underwent biopsy (including the patient above). Each of the kidney biopsies showed AIN (Table 1). 

### 3.3. Dialysis Therapy

Two patients required dialysis for uremic symptoms. Both underwent hemodialysis for a period of 10 days. Both underwent kidney biopsies which confirmed AIN. 

### 3.4. Steroid Therapy

One patient with biopsy confirmed AIN required steroid therapy for six months for complete recovery of renal function [8]. 

### 3.5. Outcome

The mean duration of hospitalization was 7.4 ±5.5 days. All patients recovered renal function with normalization of serum creatinine to 0.71 ±0.15 mg/dL (range 0.5-1.0, p <0.01) and mean baseline eGFR of 134 ± 26.2 mL/min (range 89-177, p < 0.01). However, the duration from onset to normalization of serum creatinine was 37 ± 42 days; indicating many patients had abnormal renal function for a prolonged period (Table 2). 

**Table 2:** Outcome of 15 inpatients with NSAID induced AKI.

Patient	Peak Creatinine (mg/dL)	eGFR (ml/min)	Days to Normalization	Baseline Creatinine (mg/dL)	eGFR baseline (ml/min)	Length of Stay(days)	Dialysis	Steroids
1	3.4	26	20	0.8	110	4	N	N
2	3.1	40	27	0.9	138	6	N	N
3	1.6	54	14	0.5	174	2	N	N
4	1.8	50	16	1.0	89	3	N	N
5	13	6	33	0.8	99	21	Y	N
6	1.2	77	9	0.6	153	3	N	N
7	3.6	26	16	0.7	132	3	N	N
8	1.4	64	51	0.6	149	7	N	N
9	1.5	58	4	0.9	97	6	N	N
10	15.3	7	152	0.7	152	17	Y	Y
11	3	31	8	0.7	132	11	N	N
12	1.4	49	7	0.5	136	6	N	N
13	4.8	17	105	0.6	133	9	N	N
14	2.9	33	23	0.7	138	3	N	N
15	3.3	38	75	0.7	177	10	N	N

On follow up, none of the patient developed a relapse of any kind of unrelated renal disease. All of them did have repeat UA on multiple occasions. At the last followup, all patients had a normal UA.

## 4. Discussion

We describe fifteen inpatients that were previously healthy without a history of previous kidney conditions and developed AKI. All of our cases were non-oliguric. Two of the patients required hemodialysis. Of the fifteen patients only one had nephrotic syndrome clinically and the patient’s kidney biopsy confirmed AIN and minimal change disease. The patient’s nephrotic syndrome resolved spontaneously without steroids. Fourteen out of fifteen patients had at least doubling of the serum creatinine. Only one of our patients required steroid therapy as rest had decreasing serum creatinine prior to discharge from hospital. Steroid therapy has been used in AIN successfully, though no controlled trials have demonstrated consistent benefits [7,8]. The one patient who required steroid therapy also required hemodialysis for ten days and took six months to return to baseline renal function [8]. All patients recovered renal function; however, many patients had abnormal serum creatinine for prolonged periods; eight patients required weeks to months to normalize their renal function. None of the patients developed a relapse of any kind of unrelated renal disease. At the last followup, all patients had a normal UA.

NSAIDs are a known cause of AKI, yet as an entity they may remain under diagnosed because the kidney failure is often moderate, asymptomatic, transitory, and non-anuric [2,10]. Under normal physiological conditions, renal blood flow is either independent of prostaglandin synthesis or, under certain circumstances, there is activation of the renin-angiotensin system. When circulating vasoconstrictors are released and to maintain renal blood flow, counter-regulatory prostoglandins are released [10]. NSAIDs exert antipyretic, analgesic, and anti-inflammatory effects by reducing vasodilatory prostaglandin biosynthesis [2]. AKI can occur from NSAID induced renal interstitial inflammation, resulting in AIN.

Tubulointerstitial nephritis is an inflammatory pathology of renal interstitium and tubules with acute damage, edema, and can potentially heal with interstitial fibrosis [1,6,11]. Clinically, AIN resembles ATN though sometimes signs such as rash, eosinophilia, and eosinophiluria can be present [1,6,11]. Over 2/3 of AIN is drug induced [1,6,11]. It is important to recognize when AIN may be due to a drug because prolonged injury may cause permanent scarring, and withdrawal of offending agent is obligatory [1,6,11]. 

NSAID nephropathy caused by hypersensitivity can cause a reaction that is milder than in drug-induced AIN, probably because the offending drug inhibits the inflammatory reaction it has started itself [6,7,8,11]. Proteinuria can be a response to lymphokines production because of immunological responses [11]. A stronger allergic reaction results in AKI with minimal proteinuria. On the other hand, immunocompetent cells can develop to produce lymphokines and lead to heavy proteinuria. Immune complexes are formed secondary to the increased glomerular permeability due to a hyperactive immune system [11].

AKI is manifested by rising serum creatinine and the majority of NSAID induced AKI cases are mild and non-oliguric, *i.e.,* urine output of >1 mL/kg/hour [6,7]. Unless serial serum creatinine values are obtained, AKI can be missed [6,7]. Prompt diagnosis of NSAID induced AKI with prompt discontinuation of the offending agent will usually reverse the condition within one week, typically within 72 to 96 hours [5]. If for any reason there is a delay in linking use of NSAIDs to AKI, nonwithdrawal of NSAIDs will continue ongoing damage leading to substantial loss of renal function up to the point of requiring dialysis support [12]. In inpatient settings, clinicians tend to recheck serial creatinine if there is oliguria. However, children with microscopic hematuria and/or proteinuria with flank pain but a negative urine culture are a group where serial creatinine estimations are highly recommended. 

NSAIDs are easily available over-the-counter in United States. The main indication for their use is pain or fever. Most of the children described in our case series were between the ages of fourteen and nineteen, and the majority admitted to taking pain killers without medical supervision. The fact they were not feeling well and did not consume enough fluids must have aggravated the renal side effects of NSAIDs. Other studies have also reported AKI after treatment with NSAIDs in children with compromised intravascular volume [3,4,6,10]. It is noted in these studies that moderate volume depletion caused by diarrhea and vomiting can aggravate the renal toxicity of NSAIDs. 

Other researchers have found that patients who were administered a high daily dose of NSAIDs had a greater risk of developing AKI than patients who were administered low to medium doses [13]. Researches have also found that there is a 4-fold increase in risk of AKI associated with NSAID use that was dose dependent during the first month of chronic usage of NSAIDs [14].

Due to the retrospective nature of our study, it has limitations. Though all children admitted to taking NSAIDs, such as ibuprofen and naproxen, the exact dose and duration could not be scientifically confirmed. Similar dilemma has been documented by other researchers investigating the exact relationship between dose of NSAIDs consumed and AKI [2,3,6]. Authors have described the amount of NSAIDs ingested as “the dosage within therapeutic limits” and “clinically prescribed limits” [2,3,6].

## 5. Conclusions

In conclusion, NSAIDs pose significant risk of renal damage and the development of AKI due to NSAIDs is under recognized. As OTC products are usually regarded safe and inadequate monitoring of side effects in children consuming NSAIDs suffering from intravascular volume contraction is a real danger. Controlled trials with NSAIDs *vs*. non-NSAIDs for antipyretic/analgesic usage are needed to evaluate exact incidence of AKI in children due to NSAIDs. Physicians need to use caution when treating fever with NSAIDs in younger children with abnormal creatinine. Physicians should discuss the risks of administering OTC NSAIDs to children. Awareness of the dangers of using NSAIDs could reduce the episodes of nephrotoxicity. 
